# Gene expression of the two developmentally regulated dermatan sulfate epimerases in the *Xenopus* embryo

**DOI:** 10.1371/journal.pone.0191751

**Published:** 2018-01-25

**Authors:** Nadège Gouignard, Tanja Schön, Christian Holmgren, Ina Strate, Emirhan Taşöz, Franziska Wetzel, Marco Maccarana, Edgar M. Pera

**Affiliations:** 1 Department of Laboratory Medicine, Lund Stem Cell Center, Lund University, Lund, Sweden; 2 Department of Experimental Medical Science, Lund University, Lund, Sweden; Laboratoire de Biologie du Développement de Villefranche-sur-Mer, FRANCE

## Abstract

Chondroitin sulfate (CS)/dermatan sulfate (DS) proteoglycans are abundant on the cell surface and in the extracellular matrix and have important functions in matrix structure, cell-matrix interaction and signaling. The DS epimerases 1 and 2, encoded by *Dse* and *Dsel*, respectively, convert CS to a CS/DS hybrid chain, which is structurally and conformationally richer than CS, favouring interaction with matrix proteins and growth factors. We recently showed that *Xenopus Dse* is essential for the migration of neural crest cells by allowing cell surface CS/DS proteoglycans to adhere to fibronectin. Here we investigate the expression of *Dse* and *Dsel* in *Xenopus* embryos. We show that both genes are maternally expressed and exhibit partially overlapping activity in the eyes, brain, trigeminal ganglia, neural crest, adenohypophysis, sclerotome, and dorsal endoderm. *Dse* is specifically expressed in the epidermis, anterior surface ectoderm, spinal nerves, notochord and dermatome, whereas *Dsel* mRNA alone is transcribed in the spinal cord, epibranchial ganglia, prechordal mesendoderm and myotome. The expression of the two genes coincides with sites of cell differentiation in the epidermis and neural tissue. Several expression domains can be linked to previously reported phenotypes of knockout mice and clinical manifestations, such as the Musculocontractural Ehlers-Danlos syndrome and psychiatric disorders.

## Introduction

Chondroitin sulfate (CS)/dermatan sulfate (DS) is a linear polysaccharide that is covalently attached to core proteins of proteoglycans and widely distributed both at the cell surface and in the extracellular matrix [1, 2]. CS/DS occurs in many invertebrates and vertebrates [3] and is involved in a range of biological functions, including the upbuilding of the extracellular matrix, cell signaling, wound healing, regeneration, and anti-coagulation [4, 5]. CS/DS is composed of alternating units of a hexuronic acid, i.e. either D-glucuronic acid (GlcA) or L-iduronic acid (IdoA), and the aminosugar N-acetyl-D-galactosamine. After synthesis of the chondroitin backbone, two DS-epimerases convert GlcA into IdoA by epimerization of the C5-carboxyl group of GlcA [6, 7], leading to the formation of hybrid CS/DS chains with a varying IdoA content. The IdoA-containing units can form long blocks or be interspersed as single moieties among unmodified GlcA-containing units. C5-epimerization and subsequent O-sulfation provide CS/DS chains with considerable structural variability and flexibility that allows interaction with matrix proteins and growth factors.

The formation of IdoA is catalyzed by DS-epi1 and DS-epi2, which are encoded by *Dse* and *Dse-like* (*Dsel*), respectively [6, 7]. Loss-of-function of these DS epimerases leads to severe consequences in vertebrates, indicating that IdoA residues in CS/DS play an important role in life. Homozygous missense mutations in *DSE* cause the musculocontractural type of Ehlers-Danlos syndrome (MC-EDS), a connective tissue disorder with congenital malformations and progressive fragility-related complications [8, 9]. Human *DSEL* (C18orf4) has been genetically linked to bipolar disorder [10] and early-onset major depressive disorder [11]. *Dse* knockout mice exhibit skin fragility that is caused by fewer IdoA residues in the CS/DS chains of the small leucine-rich proteoglycans decorin and biglycan, which leads to altered assembly of collagen fibrils in the hypodermis and dermis [12, 13]. On the other hand, no morphological and histological abnormalities have been observed in a mouse null mutant of *Dsel* [14]. Double knockout mice, entirely devoid of IdoA in their CS/DS, die around birth [15]. We recently showed that DS-epi1 has an important function in neural crest specification and cell migration in *Xenopus* embryos [16]. Single IdoA moieties in CS/DS polysaccharide chains are important for neural crest cells to adhere to fibronectin. The resemblance of the craniofacial defects in DS-epi1-deficient embryos and craniofacial anomalies in MC-EDS patients led us to suggest that MC-EDS might manifest developmental disturbances of the neural crest and therefore should be added to the list of neurocristopathies [17]. An open question is whether other congenital defects in MC-EDS might be associated with a role of DS-epi1 in the embryo. Another conundrum is how DS-epi2 malfunction might contribute to psychiatric disorders.

Although knockout mice point to an important function of CS/DS chains in early development [12, 15], the topological distribution of the chains in the embryo remains elusive. Despite that the expression of CS/DS-modifying enzymes has been presented in zebrafish [18] and of DS-biosynthetic enzymes in *Xenopus* neural crest cells [16], a comprehensive investigation of *Dse* and *Dsel* expression during embryonic development has not been performed yet. Here, we report that the two genes have similar but not identical expression patterns in the developing *Xenopus* embryo. The expression domains are consistent with locations of phenotypic alterations in mutant mice. They also coincide with clinical manifestations in human MC-EDS patients and might substantiate the genetic associations with psychiatric disorders.

## Materials and methods

### Constructs and RNA synthesis

A full-length cDNA sequence of *Xenopus laevis Dsel*.*L* with 86 nucleotides of the 5’ untranslated region (5’UTR), the open reading frame and 8 nucleotides of the 3’UTR was PCR-amplified from pCR-XL-TOPO-*Dsel*.*L* [16], subcloned into the pCS2 vector and completely sequenced. *X*. *laevis* cDNA fragments of *Dse*.*S* (nucleotides 828–1823 from GenBank accession number KU877109) and *Dsel*.*L* (nucleotides 2582–3608 from GenBank accession number KU877110) were PCR amplified from pCS105-*Dse*.*S* (Osada/Taira NBRP *Xenopus* ANE library, clone ID: XL487g09ex; [19]) and pCR-XL-TOPO-*Dsel*.*L* [16], subcloned into pCR4-TOPO (Thermo Fisher Scientific) and completely sequenced.

Sense mRNA for microinjection was synthesized using the mMessage Machine Kit (Ambion). Full-length cDNA plasmids were linearized with a restriction enzyme and transcribed with an RNA polymerase as follows: pCS105-*Dse*.*S* (BstXI, Sp6), pCS2-*Dsel*.*L* (NotI, Sp6) and pCS2-*nlacZ* (NotI, Sp6; a kind gift of Dr. Tomas Pieler, Univ. Göttingen, Germany).

For *in vitro* transcription, plasmids were linearized and antisense RNAs transcribed as follows: pCR4-TOPO-*Dse*.*S* (NotI, T3; also used in [16]), pCR4-TOPO-*Dsel*.*L* (SpeI, T7; also used in [16]), *En2* (XhoI, T7; [20]), *Foxg1* (XhoI, Sp6; [21],*Pax3* (BglII, Sp6, [22]), *Snai2* (ClaI, Sp6; [23]), *Twist1* (EcoRI, T7; [24]), *Xag1* (NcoI, Sp6, a kind gift of Dr. Tomas Pieler, Univ. Göttingen, Germany). Sense RNA of pCR4-TOPO-*Dse*.*S* (Sp6, T7) was generated for *in situ* hybridization.

### Frog handling

All *Xenopus* experiments reported in this study have been approved by the Lund/Malmö regional ethical committee (M140-14). Adult sexually mature *Xenopus laevis* frogs were purchased from Nasco (Fort Atkinson, WI, USA). Female and male frogs were housed in separate racks with a recirculation system (Tecniplast, Italy). A total of 20 female and 5 male frogs were used for this study. Ovulation was stimulated by injection of 750 units of human chorionic gonadotropin (Sigma CG10) into the lymph sac of a female frog. Female frogs were re-used for egg collection after a recovery period of at least 3 months. For testis isolation and *in vitro* fertilization, male frogs were sacrificed by incubation for 10 min in 0.05% benzocaine (Sigma E1501) and decapitation.

### Embryo manipulations, whole-mount *in situ* hybridization and vibratome sections

Embryos were prepared, microinjected, cultured and analyzed by Red-Gal staining and whole-mount *in situ* hybridization as described in a previous publication [25]. For whole-mount *in situ* hybridization, treatment with Proteinase K (Ambion AM2546) and a hybridization temperature of 65°C were used. Stained embryos were sectioned with a surgical blade (Ref. 0301; Swann-Morton, Sheffield, U.K.). For vibratome sections, stained embryos were equilibrated in PBS containing 4.44 mg/ml gelatin (Merck, 1.04078) and 0.27 g/ml bovine serum albumin (Sigma, A3912). After equilibration, embryos were mounted by solidifying the solution with 0.075 volume glutaraldehyde (25% stock, Merck, 1.04239) and cut as 50 μm sections with a Leica VT 1200S vibratome (Leica Microsystems AB, Sweden).

### RNA isolation and gene expression quantification by RT-qPCR

Total RNA from 10 embryonic explants per sample was extracted using the RNeasy Mini Kit (Qiagen) and reverse transcribed to cDNA (Quantitect Reverse Transcription Kit, Qiagen). Real-time quantitative PCR was run using the FAST SYBR Green Master Mix (Ambion Life Technologies) and the Mx3005P qPCR system (Stratagene) with standard cycling parameters. Fold expression values were calculated using the 2^-ΔCt^ formula, using the housekeeping gene *eEF1A1* as control. The sequences of the primers (Invitrogen) have been described previously [16].

## Results

### Antisense RNA probes specifically detect *Dse* and *Dsel* mRNAs in *Xenopus* embryos

**Fig 1:**
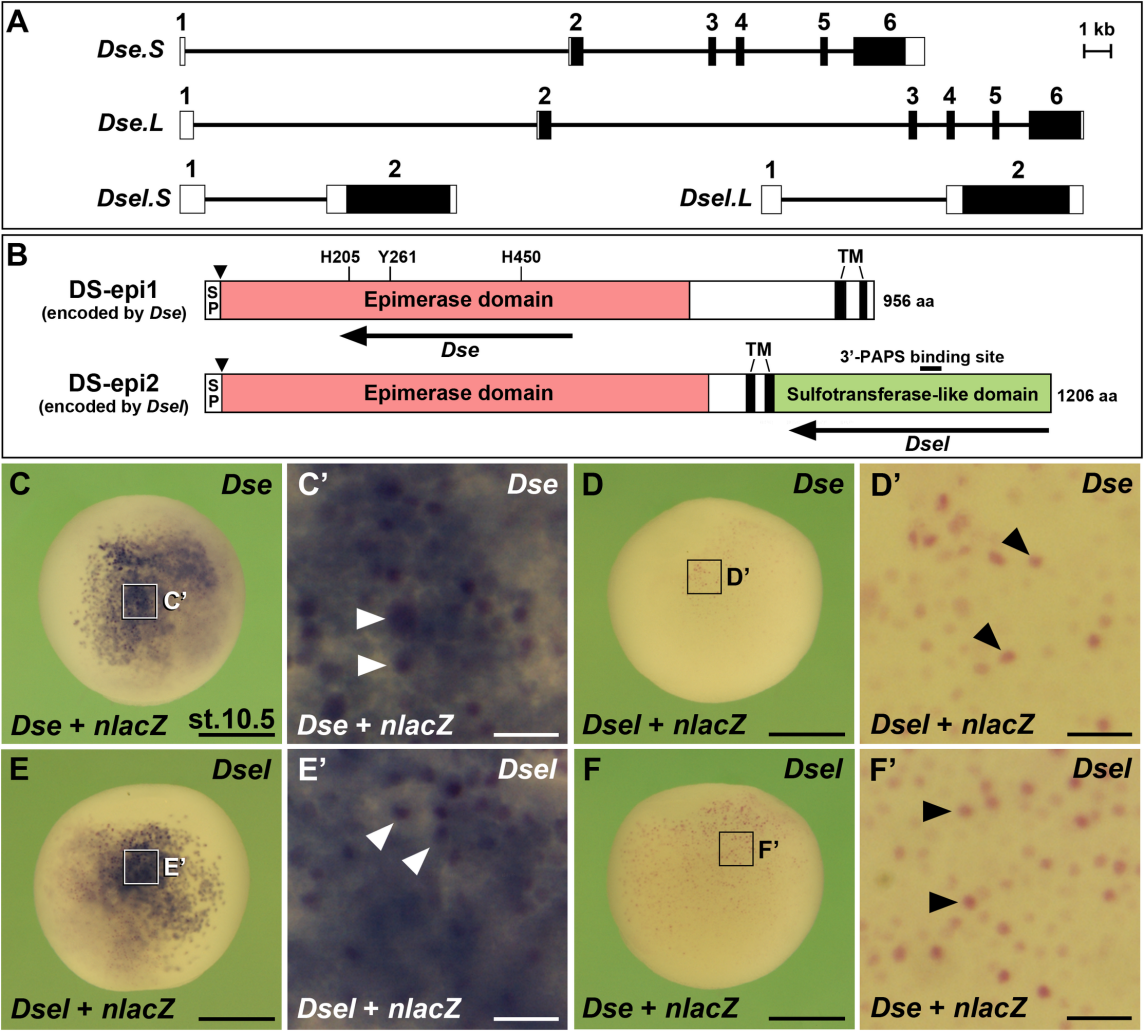
Genomic organization, protein structure and probe specificity of *Dse*/DS-epi1 and *Dsel*/DS-epi2 in *X*. *laevis* embryos. **(A)** Genomic structures of the *Dse* and *Dsel* homeologs. Rectangles with numbers show exons and intervening lines demarcate introns. Filled boxes indicate open reading frames. Accession numbers of the *Xenopus laevis* genomic DNA / mRNA sequences are: DS-epi1.S, NC_030733 / KU877109; DS-epi1.L, NC_030732 / XM_018263281; DS-epi2.S, NC_030735 / XM_018223616; DS-epi2.L, NC_030734 / KU877110. **(B)** Overall protein structure of DS-epi1 and DS-epi2, which are encoded by *Dse* and *Dsel*, respectively. SP, cleavable signal peptide; TM, transmembrane domain. The arrows represent the antisense RNA probes of *Dse* and *Dsel* that were used for the *in situ* hybridization. **(C-F’)** Whole-mount *in situ* hybridization of early gastrula embryos in lateral view. The *Dse* probe detects injected *Dse* mRNA (C,C’) but not *Dsel* mRNA (D,D’). The *Dsel* probe specifically targets injected *Dsel* mRNA (E-F’). Arrowheads depict cells that received co-injected *nlacZ* mRNA as a lineage tracer (red nuclei). Each synthetic mRNA was injected at a dose of 100 pg into the animal pole of a single blastomere at the 4-cell stage. Scale bars are 500 μm (C-F) and 50 μm (C’-F’).

We recently isolated full-length cDNA clones of *Dse* and *Dsel*, for which each two homeologous copies exist in *X*. *laevis* [16]. *Dse*.*S* and *Dse*.*L* are located on chromosomes 5S and 5L, respectively, while *Dsel*.*S* and *Dsel*.*L* are on the 6S and 6L chromosomes (NCBI, International *Xenopus* Sequencing Consortium, UC Berkeley, USA). The genomic loci of *Dse*.*S* relative to *Dse*.*L* and of *Dsel*.*S* relative to *Dsel*.*L* are 18% and 14% smaller, respectively (Fig 1A), likely as a consequence of small-scale deletions due to intra-chromosomal rearrangements that are more common to the S (short) than the L (long) subgenome in *X*. *laevis* [26]. The *Dse* gene consists of six exons with an open reading frame that spans from exon 2 to exon 6, whereas the *Dsel* gene has two exons with a continuous open reading frame on the second exon. This genomic structure is conserved in human *DSE* and *DSEL* [7]. At the nucleotide level, the coding regions of the short and long homeologous *X*. *laevis* copies are 93% (*Dse*) and 95% (*Dsel*) identical. At the amino acid level, their identities are 94% (DS-epi1 encoded by *Dse*) and 96% (DS-epi2 encoded by *Dsel*) [16]. The amino acid similarity between the S and L copies of each protein is 97%. *Xenopus* DS-epi1 and DS-epi2 encompass 956 and 1206 amino acids, respectively (Fig 1B; [16]). Like their human counterparts [7], they share a cleavable signal peptide, a ~700 amino acid domain of an active epimerase domain and two transmembrane segments. A comparison of the *X*. *laevis* amino acid sequences reveals an identity of ~50% in the common epimerase domains of DS-epi1 and DS-epi2 (S1 Fig; [16]). Three amino acids in the epimerase domain that are required for its catalytic activity in human DS-epi1 (H205, Y261, H450; [27]) are conserved in each homeologous copy of the *X*. *laevis* DS-epi1 and DS-epi2 enzymes. In addition, DS-epi2 contains a carboxyterminal domain with homology to several carbohydrate sulfotransferases, including a short signature of the 3’-PAPS (3’-phosphoadenosine-5’-phosphosulfate)-binding site. This homology makes plausible a sulfotransferase activity of DS-epi2 although conclusive data are not available.

In order to analyze the gene expression of *Dse* and *Dsel*, we generated ~1 kb antisense RNA probes against the epimerase domain of DS-epi1 and the sulfotransferase-like domain of DS-epi2 (Fig 1B). Given the sequence homology of both genes in the epimerase domain, we first tested the specificity of the *Dse* probe in *Xenopus* embryos that were microinjected with either *Dse* or *Dsel* mRNA. Our previous RT-PCR analysis showed that endogenous mRNA levels of *Dse* and *Dsel* are relatively low at the early gastrula stage [16]. Whole-mount *in situ* hybridization at stage 10.5 showed that the *Dse* probe unambiguously detected cells derived from a *Dse* mRNA-injected blastomere (Fig 1C and 1C’). In contrast, the same probe failed to bind to exogeneous *Dsel* mRNA (Fig 1D and 1D’). As expected, the *Dsel* probe bound to injected *Dsel* mRNA (Fig 1E and 1E’) and not to exogeneous *Dse* mRNA (Fig 1F and 1F’). Thus the antisense RNA probes against *Dse* and *Dsel* specifically detect their respective target mRNAs.

### Expression of the *Dse* gene

**Fig 2:**
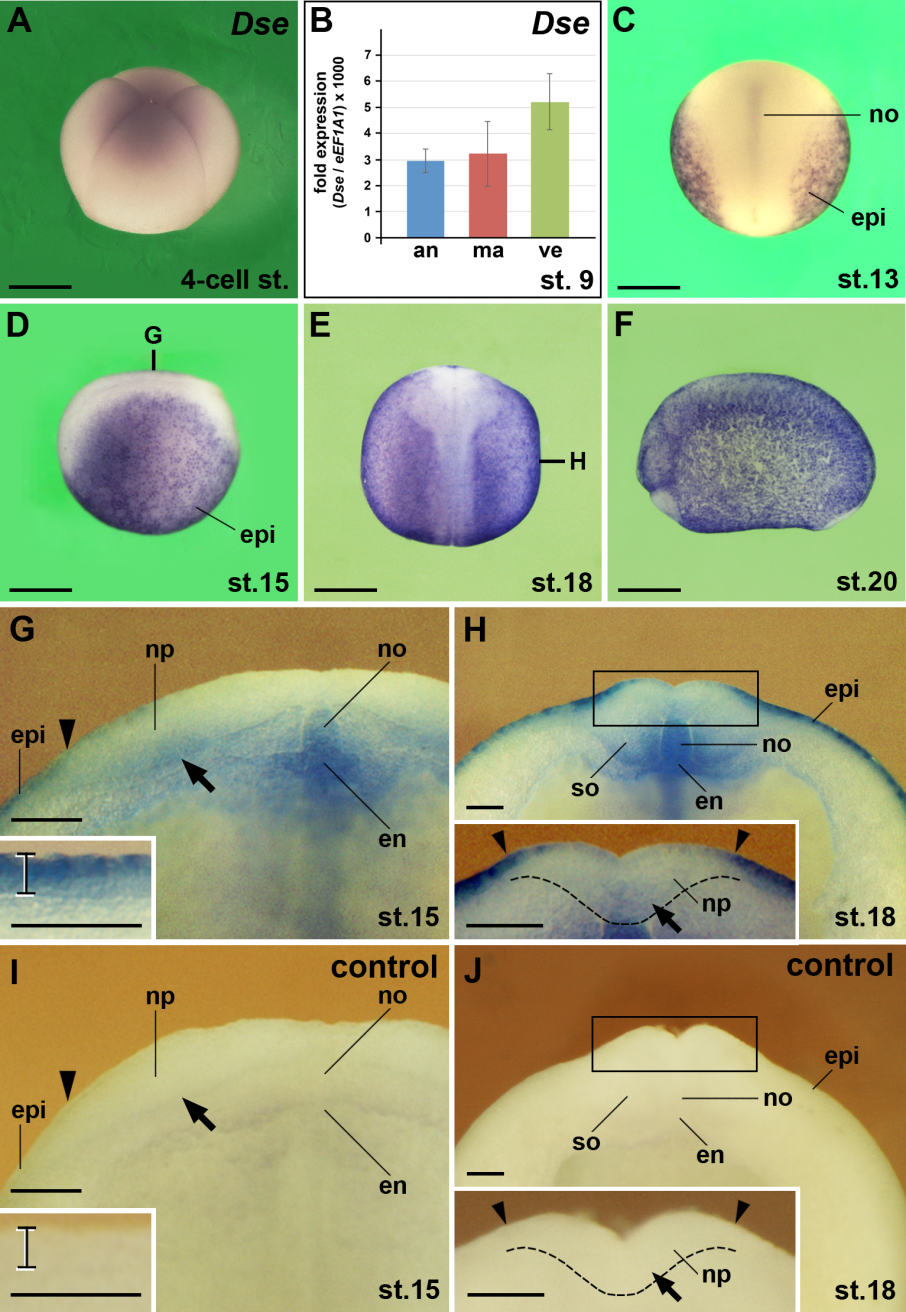
*Dse* mRNA is maternally deposited and expressed in the epidermis, neural ectoderm, notochord, somites and dorsal endoderm. *Xenopus* embryos after whole-mount *in situ* hybridization in lateral view (A,D,F), dorsal view (C,E) and transversally sectioned through the anterior trunk (G-J). **(A)** Embryo at the 4-cell stage. Weak expression of *Dse* mRNA is visible. **(B)** qPCR analysis of embryonic explants at stage 9. Note *Dse* transcripts in the animal cap, marginal zone and vegetal region. **(C)** At stage 13, *Dse* is expressed in the epidermis and notochord. **(D-F)** Neurula embryos show ubiquitous *Dse* expression in the epidermis. The bold lines indicate the levels of sections in G and H. **(G)** Embryo at stage 15. The arrowhead indicates the border between epidermis and neural ectoderm. Note *Dse* transcripts in the sensorial layer of the lateral neural plate (arrow), notochord and dorsal endoderm. The bracket in the inset depicts the epidermis with robust *Dse* expression in the outer layer and lower mRNA levels in the inner layer. **(H)** Embryo at stage 18. Note *Dse* signals in the medio-ventral somites. The inset shows *Dse* expression in the sensorial layer of the ventral neural groove (arrow). The stippled line demarcates the border between the neural plate and the underlying dorsal mesoderm. **(I,J)** No signals appear in matching sections of sibling embryos that were probed with *Dse* sense RNA as control. an, animal cap; en, endoderm; epi, epidermis; ma, marginal zone; no, notochord; np, neural plate; so, somite; ve, vegetal region. Scale bars are 500 μm (A,C-F) and 50 μm (G-J).

By whole-mount *in situ* hybridization, we detected low levels of *Dse* mRNA in 4-cell stage embryos, suggesting maternal *Dse* transcription (Fig 2A). Quantitative real time PCR (qPCR) showed *Dse* signals in animal, marginal and vegetal explants at stage 9 (Fig 2B). From (mid gastrula) stage 13 onwards, zygotic *Dse* expression was seen in the epidermis and notochord (Fig 2C–2H). A transversal hemi-section at stage 15 revealed robust *Dse* expression in the superficial layer and lower-level expression in the sensorial layer of the epidermis (inset in Fig 2G). The inner sensorial layer of the lateral neural plate showed weak *Dse* signals (Fig 2G). Additional signals were seen in the dorsal medial endoderm from which the hypochord will evolve. At (neurula) stage 18, low-level *Dse* expression shifted from the lateral to the medial (now ventral) sensorial layer of the neural groove at the level of the future mid- and hindbrain (inset in Fig 2H). *Dse* transcripts appeared in the ventro-medial edge of the somites (Fig 2H) that will give rise to the sclerotome [28]. *In situ* hybridization with a sense RNA probe did not show any signal in embryos at stage 15 and 18 (Fig 2I and 2J), confirming the specificity of *Dse* detection.

**Fig 3:**
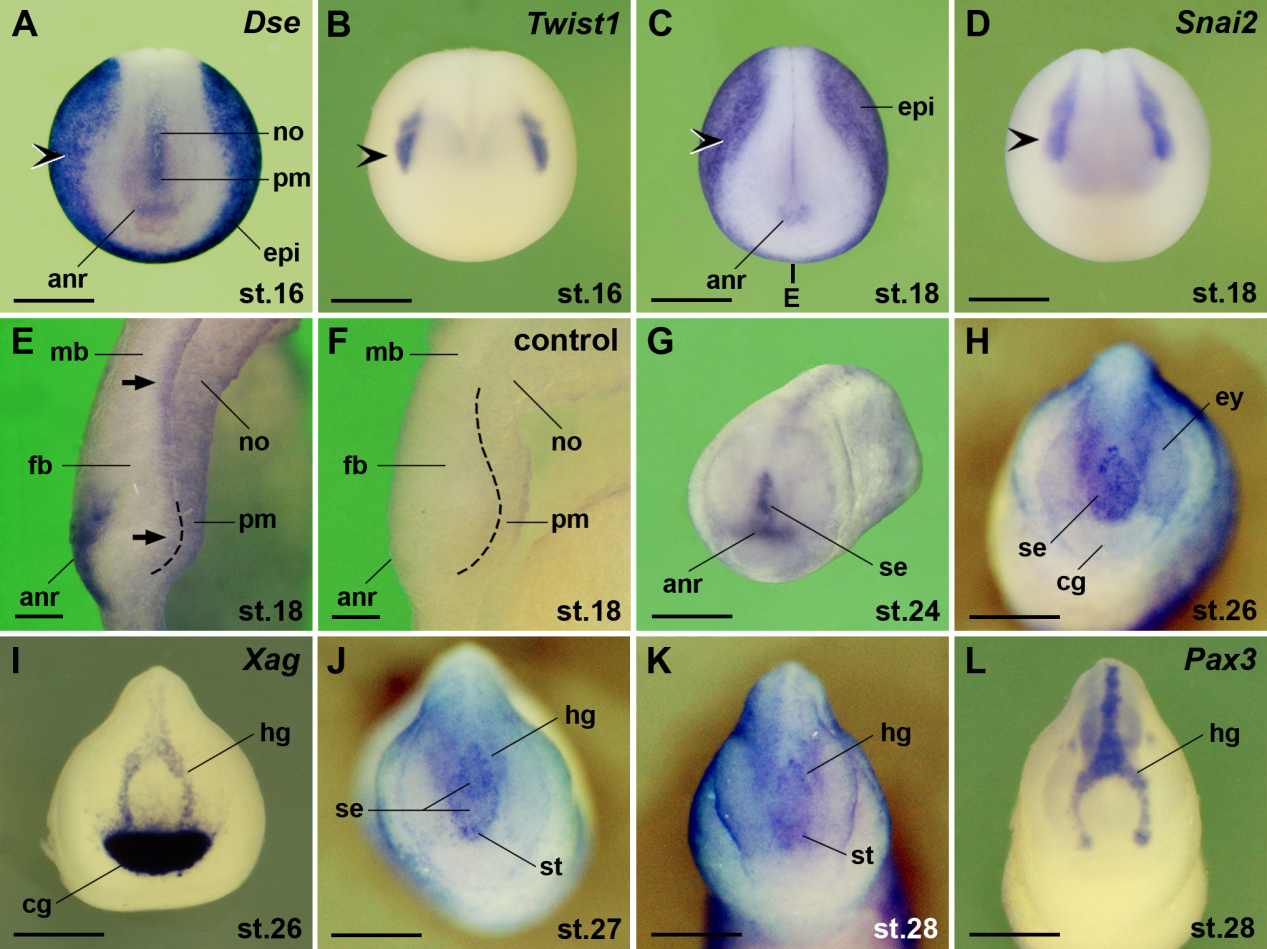
*Dse* is expressed in the pre-migratory neural crest, anterior surface ectoderm, brain, and prechordal mesendoderm. Embryos are shown in anterior view (A-D, G-L) and midsagittally sectioned through the head (E,F). **(A-D)** Early neurulae. Note *Dse* expression in the anterior neural ridge and prechordal mesendoderm. *Dse* transcripts overlap with *Twist1* and *Snai2* expression in the cranial neural crest (indented arrowheads). The bold line indicates the level of section in E. **(E)** Embryo at stage 18. *Dse* is expressed in the superficial layer of the anterior neural ridge and the inner layer of the fore- and midbrain anlage (arrows). The stippled line demarcates the border between the anterior neural plate and the underlying prechordal mesendoderm. **(F)** Absence of signals in the matching section of a sibling embryo that was probed with *Dse* sense RNA as control. **(G)** Late neurula at stage 24. *Dse* is expressed in the anterior neural ridge and derived sense plate. **(H)** Tailbud embryo at stage 26. *Dse* is transcribed in the sense plate. **(I)** *Xag* expression demarcates the cement gland and hatching gland. **(J-L)** Tailbud embryos at stages 27 and 28. Note segregation of anterior *Dse* expression into the stomodeum-adenohypophysis anlage and posterior domain that partially overlaps with the *Pax3*^+^ hatching gland. anr, anterior neural ridge; cg, cement gland; epi, epidermis; ey, eye; fb, forebrain; hg, hatching gland; mb, midbrain; no, notochord; pm, prechordal mesendoderm; se, sense plate; st, stomodeum-adenohypophysis anlage. Scale bars are 500 μm (A-D,G-L) and 50 μm (E,F).

We previously showed that *Dse* is expressed in cranial neural crest cells [16]. Here we confirm its expression in the *Twist1*- and *Snai2*-positive pre-migratory neural crest at the border between epidermis and anterior neural plate (Fig 3A–3D). In early neurulae, *Dse* transcripts appeared in the superficial layer of the anterior neural ridge and in the prechordal mesendoderm (Fig 3A, 3C and 3E). At stage 18, weak *Dse* signals were also detected in the inner layer of the forebrain and midbrain anlage (Fig 3E). The absence of signals in a sibling embryo probed with a sense RNA underscore the specificity of the *Dse* signals (Fig 3F). At stage 24, *Dse* expression extended dorsally into the sense plate (Fig 3G). The sense plate arises from the anterior neural ridge and comprises the anlage of the stomodeum, adenohypophyseal placode and olfactory placodes [29–31]. At stage 26, the expression domain of *Dse* in the sense plate (Fig 3H) was flanked by *Xag* expression in the cement gland and hatching gland (Fig 3I). *Dse* expression in the sense plate then segregated into the anterior stomodeum-adenohypophysis anlage and a posterior bifurcated domain (Fig 3J and 3K) that abutted and partially overlapped with *Pax3*^+^ hatching gland cells (Fig 3L).

**Fig 4:**
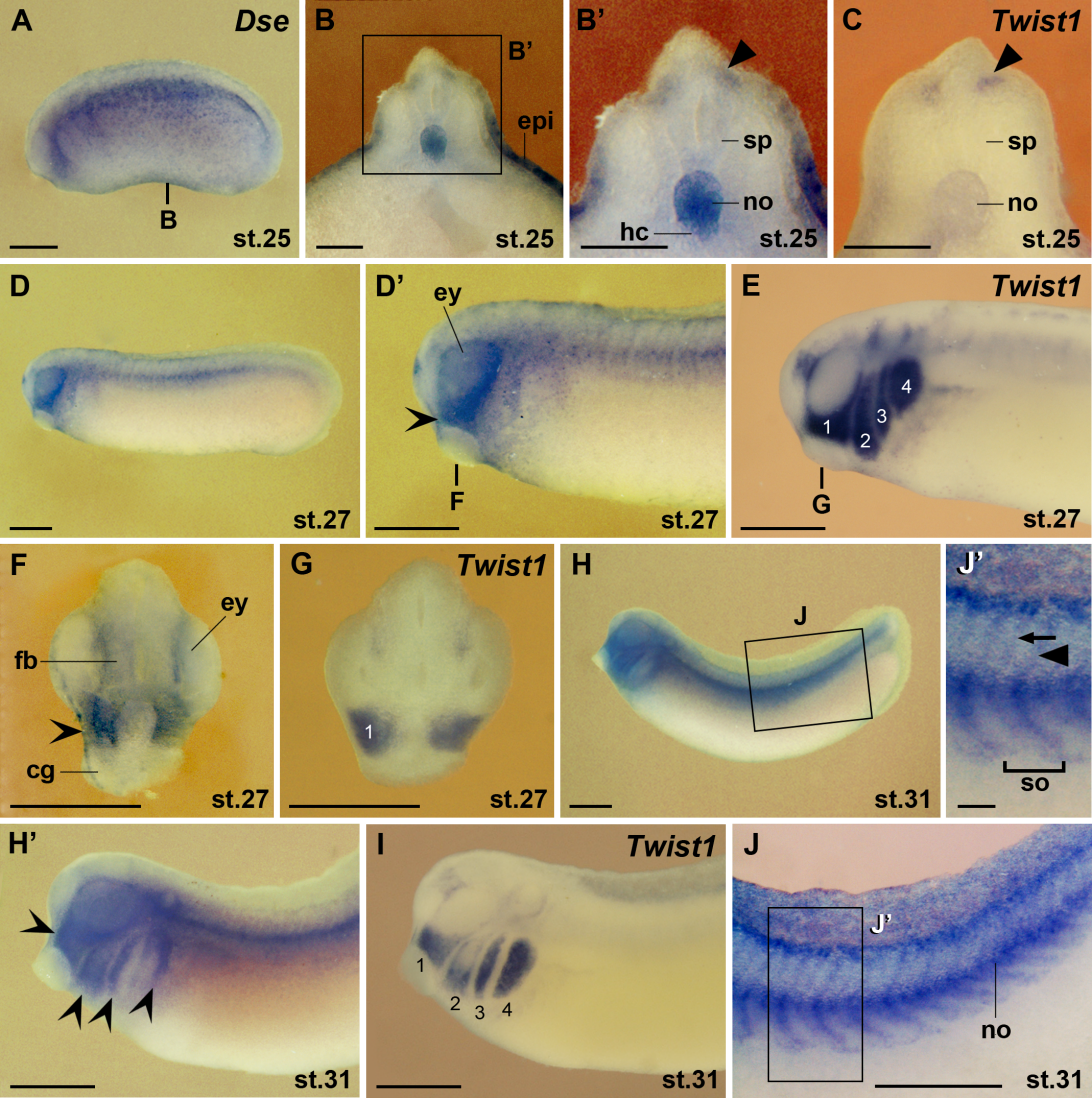
*Dse* is expressed in the eye, migratory neural crest and the hypochord. Embryos are shown in lateral view (A,D-E,H-J’), and transversally sectioned through the trunk (B-C) and head (F,G). **(A)** Embryo at stage 25. The bold line indicates the level of the section in B. **(B,B’)** *Dse* is expressed in the epidermis, notochord and hypochord. The arrowhead shows *Dse* transcripts in trunk neural crest cells adjacent to the spinal cord. **(C)** Matching section of a sibling embryo showing *Twist1* expression in trunk neural crest cells (arrowhead). **(D-I)** Tailbud embryos. The bold lines indicate the level of sections in F and G. *Dse* is expressed in the forebrain, proximal eye and *Twist1*^+^ cranial neural crest (indented arrowheads). The numbers indicate the mandibular (1), hyoid (2) and branchial neural crest (3,4). **(J,J’)** Embryo at stage 31 after removal of the epidermis. *Dse* is expressed in the spinal nerves (arrow) and migrating trunk neural crest cells (arrowhead). cg, cement gland; epi, epidermis; ey, eye; fb, forebrain; hc, hypochord; no, notochord; sp, spinal cord; so, somite. Scale bars are 500 μm (A,D-J) and 100 μm (B-C,J’).

With the extension of the primary body axis, *Dse* expression faded in the epidermis and became more robust in the notochord and underlying hypochord (Fig 4A–4B’). At stage 25, *Dse* overlapped with the *Twist1* marker in migrating trunk neural crest cells (Fig 4A–4C). At stage 27, *Dse* transcripts accumulated in the *Twist1*^+^ mandibular stream of the cranial neural crest cells and were also detected in the proximal portion of the eyes and the forebrain (Fig 4D–4G). Cranial neural crest cells arise rostral to the anterior limit of somite 4 and migrate along distinct routes as mandibular, hyoid, and branchial arch streams [32]. At stage 31, *Dse* followed *Twist1* expression in hyoid and branchial cranial neural crest cells (Fig 4H and 4I; [16]). *Dse* mRNA was also localized in the trunk neural crest cells and neural crest-derived spinal nerves that migrate between and penetrate through the somites, respectively (Fig 4J and 4J’).

**Fig 5:**
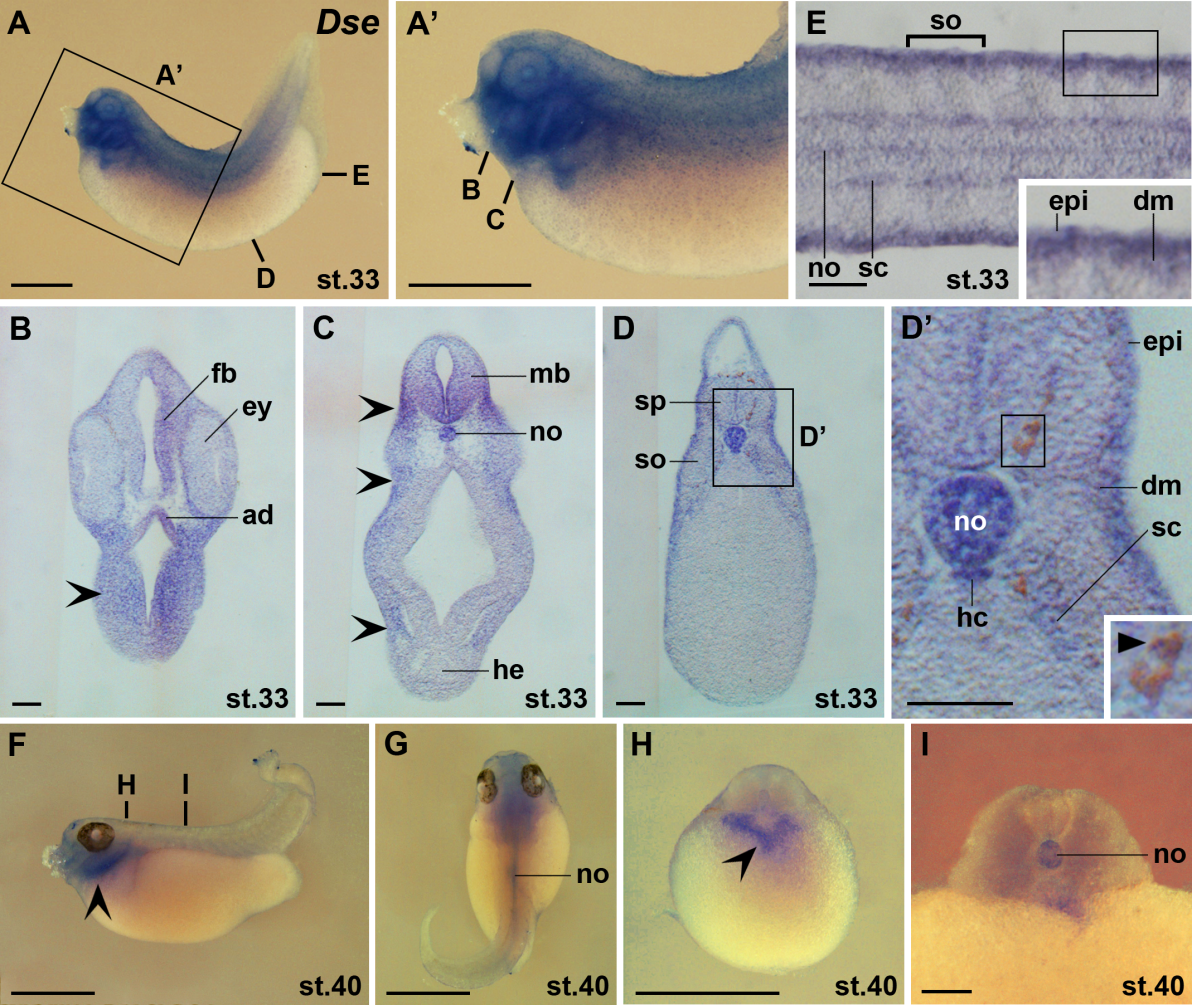
*Dse* is expressed in the adenohypophysis, melanophores, dermomyotome and sclerotome. Embryos are shown in lateral view (A,A’,F), dorsal view (G), transversal sections (B-D’,H,I) and horizontal section (E). **(A,A’)** Embryo at stage 33. The bold lines indicate the level of sections in B-E. **(B-E)** Vibratome sections showing *Dse* expression in the adenohypophysis, forebrain, ventral midbrain, epidermis, notochord, hypochord, dermomyotome, and sclerotome. Note also *Dse* transcripts in cranial neural crest cells (indented arrowheads) and melanophores (filled arrowhead). **(F-I)** Embryo at stage 40. The bold lines indicate the level of sections in H and I. *Dse* is expressed in post-migratory cranial neural crest cells (indented arrowheads) and the notochord. ad, adenohypophysis; dm, dermomyotome; epi, epidermis; ey, eye; fb, forebrain; hc, hypochord; he, heart; mb, midbrain; no, notochord; sc, sclerotome; so, somite; sp, spinal cord. Scale bars are 1 mm (A,A’,F-H) and 100 μm (B-E,I).

At stage 33, distinct *Dse* expression was observed in the ectodermal adenohypophysis, forebrain, ventral midbrain, cranial neural crest and notochord (Fig 5A–5C). More posteriorly, *Dse* expression appeared in the neural crest-derived melanophores, in the somite-derived dermomyotome and sclerotome and in the endodermal hypochord (Fig 5D and 5E). At stage 40, *Dse* transcripts were maintained in post-migratory cranial neural crest cells and in the notochord (Fig 5F–5I).

In summary, *Dse* mRNA is maternally deposited and zygotically expressed in the epidermis, anterior surface ectoderm, neural ectoderm, neural crest, prechordal mesendoderm, notochord, somites and dorsal endoderm. Later, *Dse* transcripts are found in the adenohypophysis, eyes, brain, spinal nerves, melanophores, dermomyotome, sclerotome and hypochord.

### Expression of the *Dsel* gene

**Fig 6:**
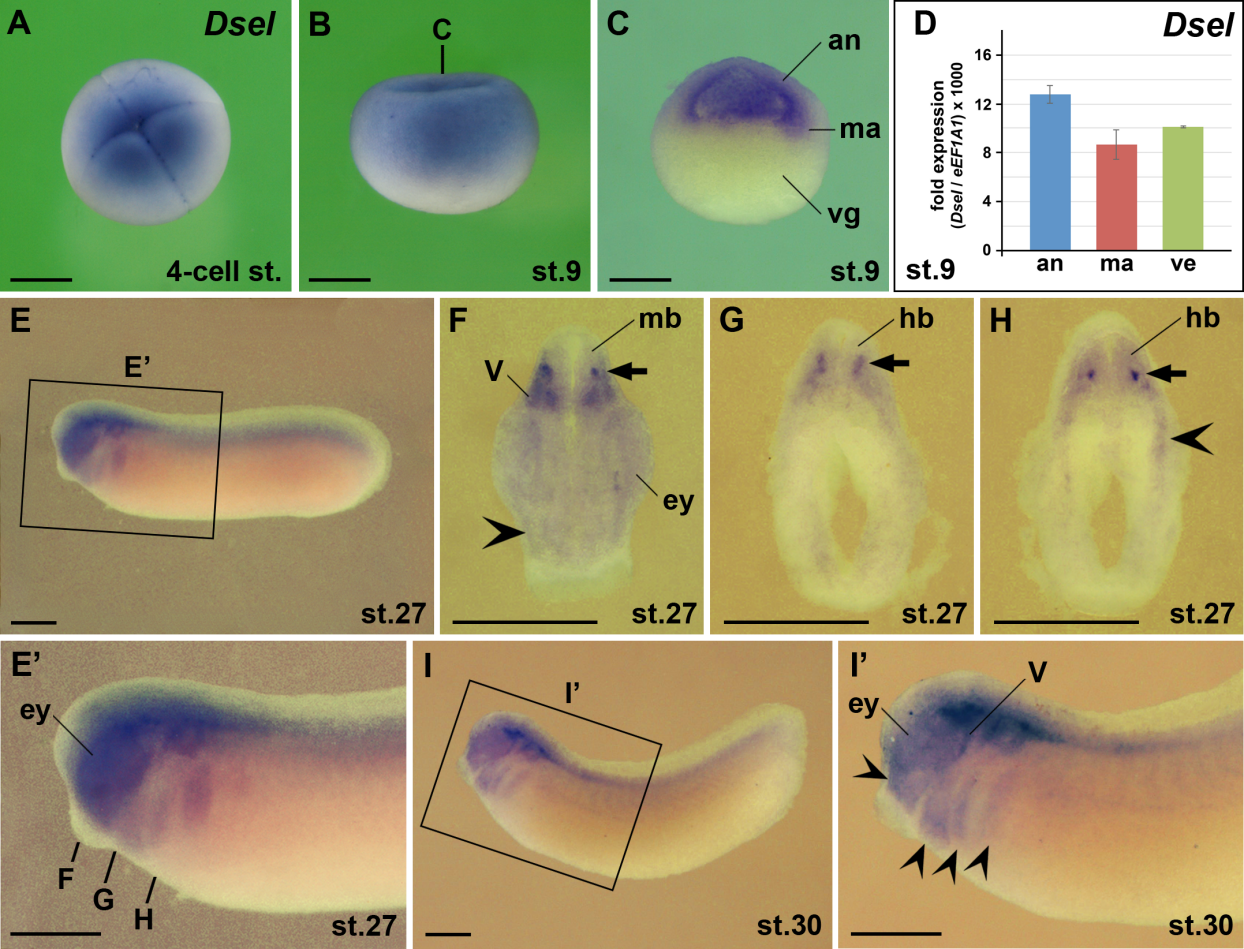
*Dsel* is expressed maternally and in the eye, brain and cranial neural crest. Embryos after whole-mount *in situ* hybridization are shown in animal view (A), lateral view (B,E,E’,I,I’), hemi-sectioned (C) and transversally sectioned (F-H). **(A)** 4-cell stage embryo. **(B,C)** Blastula embryos. The bold line indicates the level of section in C. **(D)** qPCR analysis at stage 9. Note ubiquitous expression of *Dsel* in the animal cap, marginal zone and vegetal region. **(E-I’)** Tailbud embryos. The bold lines indicate the level of sections in F-H. Note *Dsel* expression in the eye, interneurons of the mid- and hindbrain (arrows), cranial neural crest (indented arrowheads) and bilateral trigeminal ganglia. an, animal cap; ey, eye; hb, hindbrain; ma, marginal zone; mb, midbrain; V, trigeminal ganglion, vg, vegetal region. Scale bars are 500 μm.

Previous RT-PCR results showed that the maternal expression of *Dsel* is higher than that of *Dse* [16]. Our present analysis supports this conclusion. By whole-mount *in situ* hybridization, we found abundant maternal deposition of *Dsel* mRNA in 4-cell stage embryos (Fig 6A). At stage 9, *Dsel* transcripts were detected in the animal cap and marginal zone (Fig 6B and 6C). qPCR analysis showed additional *Dsel* mRNA in the vegetal region (Fig 5D), suggesting that the yolk quenches *in situ* hybridization signals in these cells. We previously demonstrated that low levels of *Dsel* transcripts are zygotically expressed in *Twist1*^*+*^ cranial neural crest cells [16]. Accordingly, we found weak *Dsel* expression in cranial neural crest cells of the mandibular, hyoid and branchial arch regions at stages 27 and 30 (Fig 6E–6H). Additional *Dsel* transcripts were observed in the eyes, distinct interneurons of the ventral midbrain and hindbrain, and in cranial neural crest-derived trigeminal (V) ganglia.

**Fig 7:**
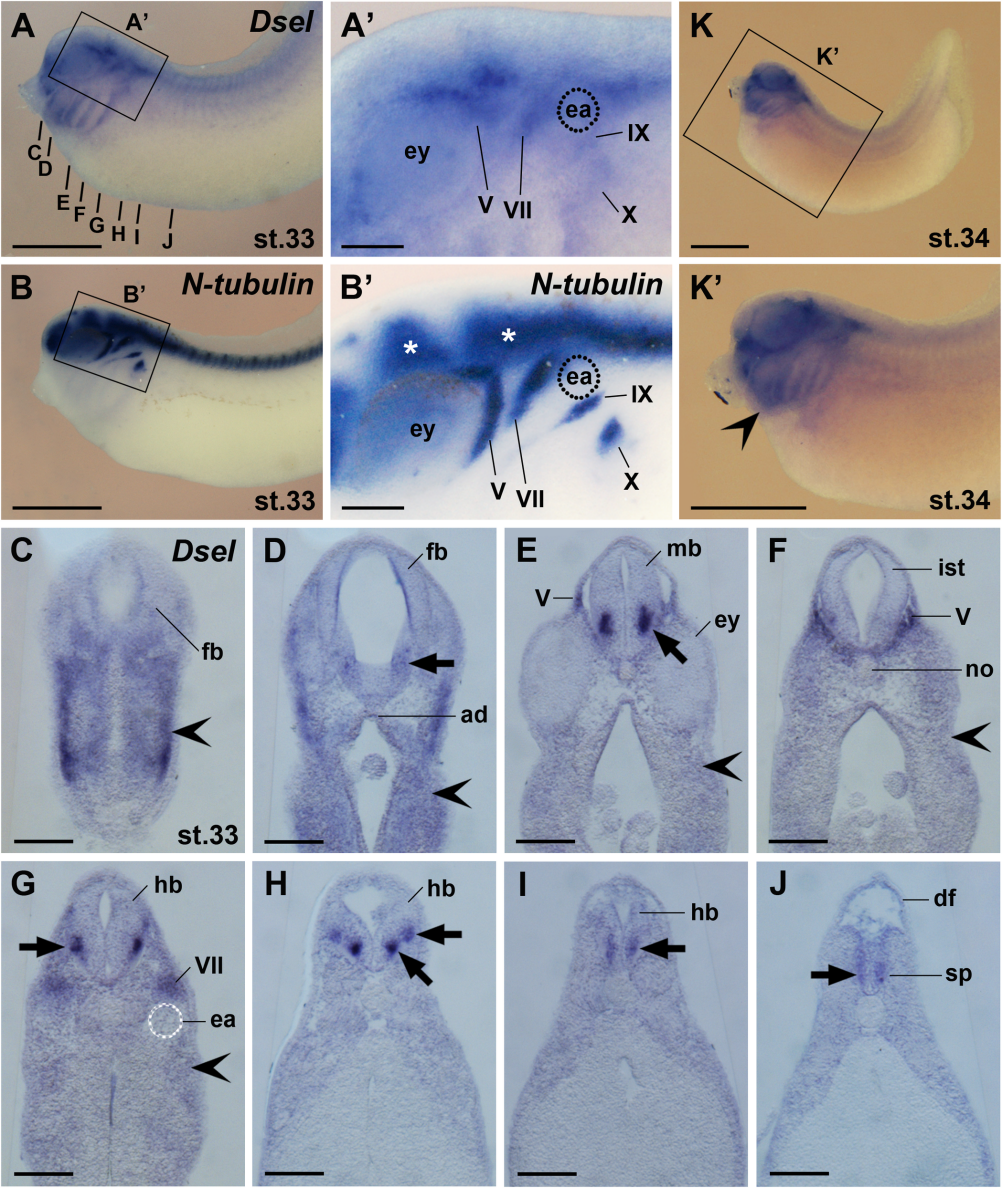
*Dsel* is expressed in differentiated neurons, cranial sensory ganglia and the spinal cord. Embryos are shown in lateral view (A-B’,K,K’) and transversal vibratome sections (C-J). **(A,A’)** Embryo at stage 33. The bold lines indicate the level of sections in C-J. Magnification in A’ depicts *Dsel* mRNA in cranial sensory ganglia: V, trigeminal ganglion; VII, geniculate ganglion; IX, petrosal ganglion; X, nodose ganglion. **(B,B’)** Sibling embryo depicting *N-tubulin* expression in the central nervous system (stars) and cranial ganglia. **(C-J)** *Dsel* is expressed in the adenohypophysis and distinct neurons (arrows) of the forebrain, midbrain, hindbrain and spinal cord. The indented arrowhead labels signals in migrating cranial neural crest cells. **(K,K’)** Embryo at stage 34. The indented arrowhead labels robust *Dsel* transcripts in post-migratory cranial neural crest cells. ad, adenohypophysis; df, dorsal fin; ea, ear; ey, eye; fb, forebrain; hb, hindbrain; ist, isthmus; IX, petrosal ganglion; mb, midbrain; no, notochord; pm, sp, spinal cord; V, trigeminal ganglion; VII, geniculate ganglion; IX, petrosal ganglion; X, nodose ganglion. Scale bars are 1 mm (A,B,K,K’) and 200 μm (A’,B’,C-J).

At stage 33, *Dsel* was maintained in cranial neural crest cells (Fig 7A and 7C–7G). *Dsel* overlapped with *N-tubulin* expression in the trigeminal (V) ganglia and three neural crest-derived epibranchial ganglia, including the geniculate (VII), petrosol (IX) and nodose (X) ganglia (Fig 7A–7B’ and 7E–7G). A more detailed analysis of histological sections showed weak *Dsel* expression in the adenohypophysis and ventrolateral forebrain (Fig 7D). More robust signals appeared bilaterally in the ventral marginal zone of the midbrain and hindbrain, where interneurons differentiate (Fig 7E, 7G and 7H). The lower expression levels in the isthmus (Fig 7F) suggest that the *Dsel*-positive interneurons in the adjacent midbrain (Fig 7E) and anterior hindbrain (Fig 7G) are non-continuous. Two distinct ventral columns of *Dsel*^+^ neurons were found on each side in the hindbrain (Fig 7H). Weaker bilateral *Dsel* expression domains appeared in the marginal zone of the posterior hindbrain and spinal cord (Fig 7I and 7J). At stage 34, more abundant *Dsel* transcripts were detected in post-migratory cranial neural crest cells of the head and branchial arches (Fig 7K and 7K’).

**Fig 8:**
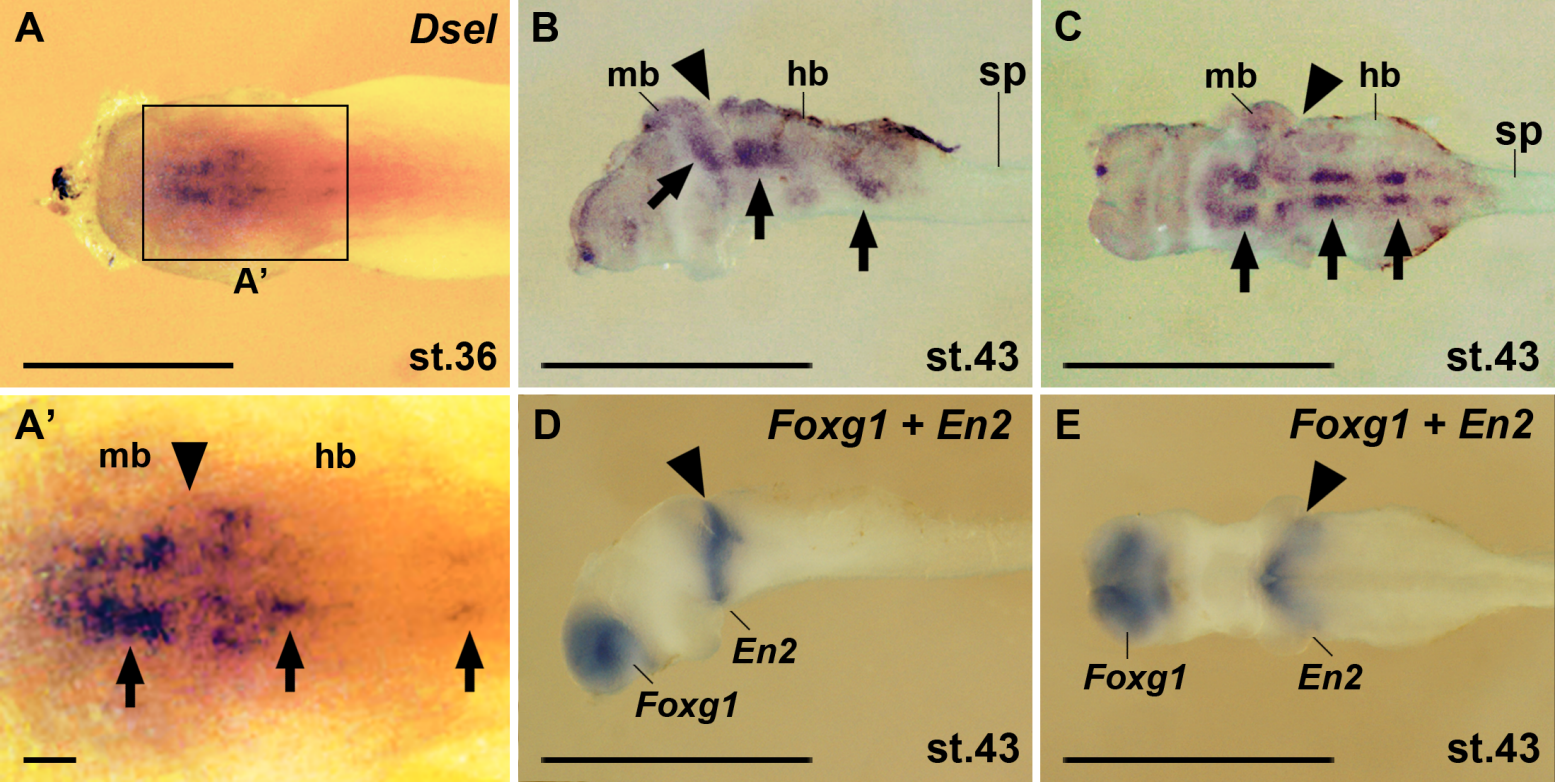
*Dsel* is expressed in the brainstem in tadpole embryos. Specimen after whole-mount *in situ* hybridization are shown in dorsal view (A,A’), lateral view (B,D) and ventral view (C,E). **(A,A’)** Embryo at stage 36. Arrows label distinct *Dsel* expression domains in the midbrain and hindbrain. The arrowhead labels the position of the midbrain-hindbrain boundary. **(B-E)** Isolated central nervous system from stage 43 embryos after removing of surrounding tissues. *Dsel* mRNA accumulates in bilateral domains in the brainstem (arrows). Expression of *Foxg1* demarcates the forebrain and *En2* the midbrain-hindbrain isthmus (arrowhead). hb, hindbrain; mb, midbrain; sp, spinal cord. Scale bars are 1 mm (A-E) and 100 μm (A’).

Swimming tadpole embryos at stages 36 and 43 showed distinct *Dsel* expression domains in the brainstem (Fig 8). Along the anteroposterior neuraxis, a single *Dsel* expression domain was seen in the ventral midbrain and at least two bilateral domains in the ventral hindbrain (Fig 8A–8C). Only weak *Dsel* expression appeared in the intervening *En2*^+^ isthmus region (Fig 8A’–8E). The *Foxg1*^+^ forebrain region and the spinal cord did not show detectable *Dsel* transcripts at these stages.

Altogether, *Dsel* is a maternally enriched gene and exhibits zygotic expression from the tailbud stage onwards in the adenohypophysis, early eyes, differentiated neurons in the brain and spinal cord, cranial neural crest, cranial sensory ganglia, prechordal mesendoderm, and hypochord.

### *Dse* and *Dsel* are co-expressed in the ventral brain, cranial neural crest cells and trigeminal ganglia

**Fig 9:**
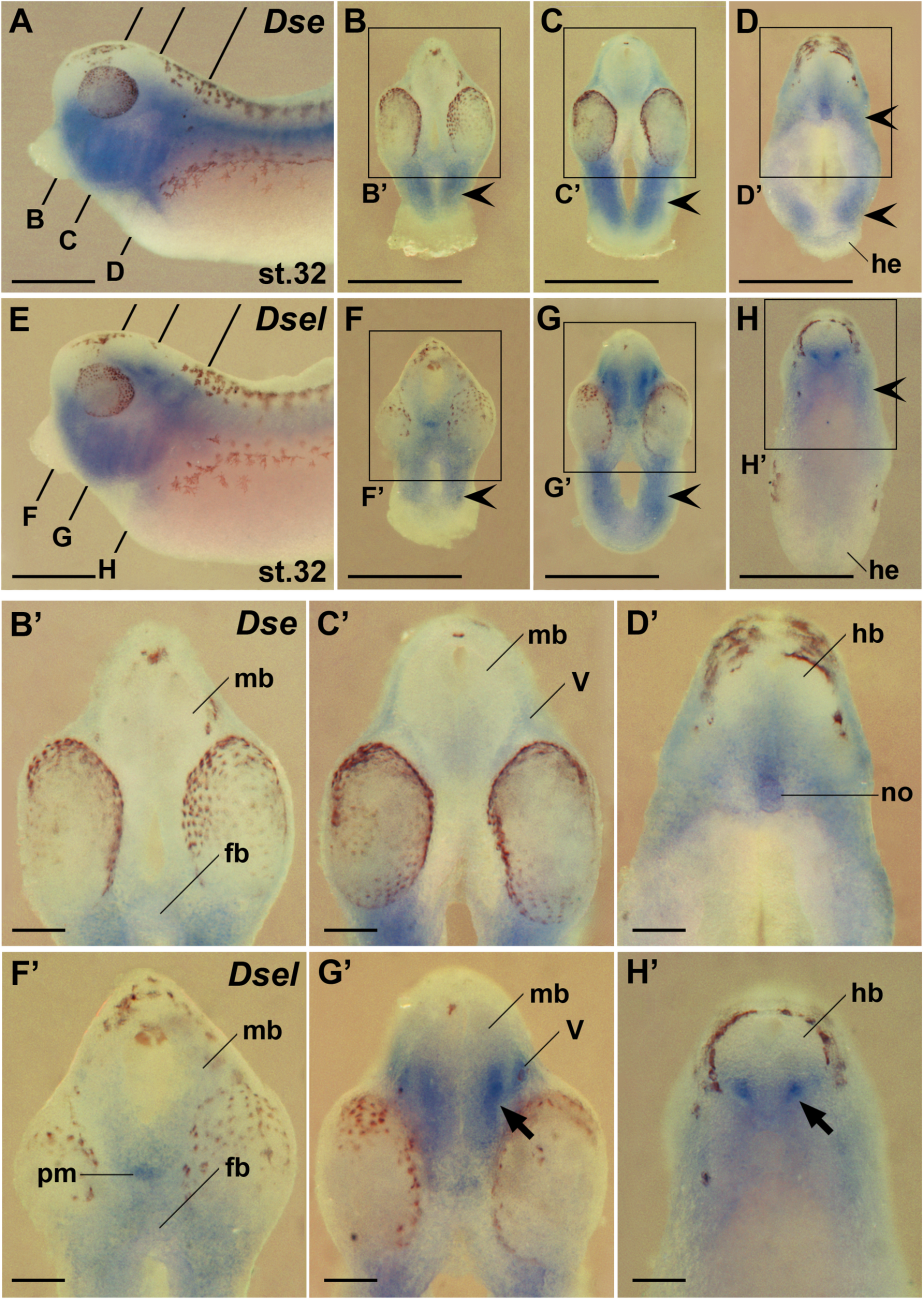
Comparison of *Dse* and *Dsel* expression in the head. Embryos at stage 32 are shown in lateral views (A,E) and transversal sections (B-D’, F-H’). **(A)** *Dse* expression. The bold lines indicate the level of sections in B-D. **(B-D’)** *Dse* expression is found in the cranial neural crest (indented arrowheads). Note ventral expression domains in the forebrain, posterior midbrain, hindbrain, and notochord. Weak expression is visible in the trigeminal ganglia. **(E)** *Dsel* expression. The bold lines indicate the level of sections in F-H. **(F-H’)** *Dsel* expression appears in the cranial neural crest (indented arrowheads) and trigeminal ganglia. Note expression domain in the prechordal mesendoderm. Apparent are low-level transcripts in the entire ventral brain and robust bilateral expression domains in the ventral marginal zone of the mid- and hindbrain (arrows). fb, forebrain; hb, hindbrain; he, heart; mb, midbrain; no, notochord; pm, prechordal mesendoderm; V, trigeminal ganglion. Scale bars are 1 mm (A-H) and 200 μm (B’-H’).

Next we compared the expression of *Dse* and *Dsel* in the head of transversally sectioned embryos after whole-mount *in situ* hybridization at stage 32 (Fig 9). The expression of both genes partially overlapped in the ventral forebrain, midbrain and hindbrain, with *Dse* mRNA (Fig 9A–9D’) being more restricted than *Dsel* mRNA (Fig 9E–9H’). Robust *Dsel* expression was observed in postmitotic ventral interneurons in the marginal zone of the mid- and hindbrain (Fig 9G’ and 9H’). *Dse* (Fig 9A–9D) and *Dsel* expression (Fig 9E–9H) largely overlapped in post-migratory cranial neural crest cells of the head mesenchyme and pharyngeal arches. *Dse* transcripts were more abundant than those of *Dsel* in posterior cranial neural crest cells adjacent to the heart (compare Fig 9A and 9D with Fig 9E and 9H). In the trigeminal (V) ganglia, *Dsel* was more robustly expressed than *Dse* (compare Fig 9A and 9C’ with Fig 9E and 9G’). Along the primary body axis, the *Dsel* gene was active in the prechordal mesendoderm (Fig 9F’), wherease *Dse* mRNA accumulated in the notochord (Fig 9D’).

### Overlapping and non-overlapping expression of *Dse* and *Dsel* in the trunk

**Fig 10:**
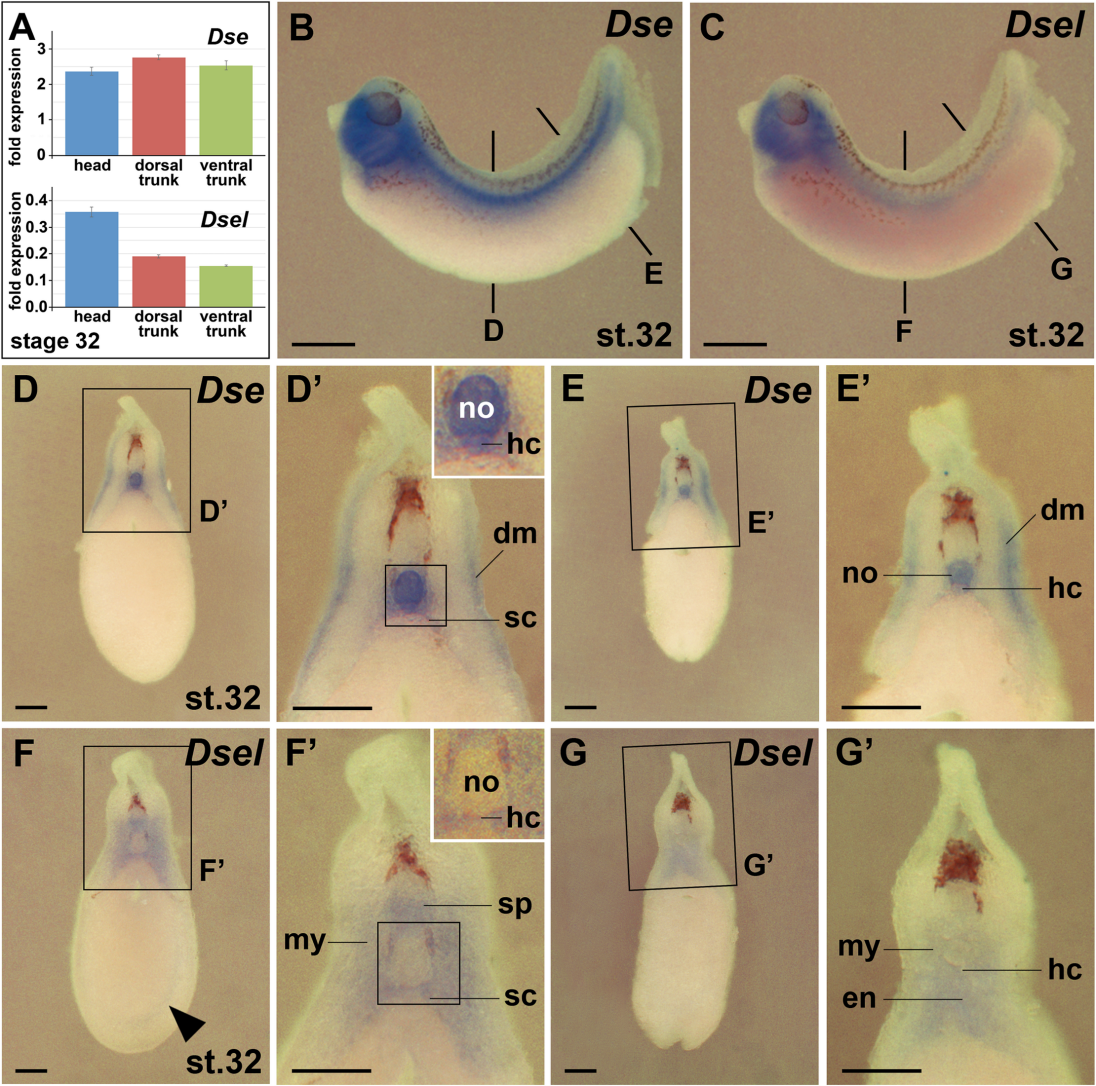
Comparison of *Dse* and *Dsel* expression in the trunk. Embryos at stage 32 are shown in lateral views (B,C) and transversal sections (D-G’) after whole-mount *in situ* hybridization. **(A)** qPCR analysis of embryonic explants at stage 32. The numbers on the y-axis indicate fold expression of (*Dse* / *eEF1A1*) x 1000 (top) and (*Dsel* / *eEF1A1*) x 1000 (bottom). Note equal distribution of *Dse* transcripts in the head, dorsal trunk and ventral trunk. *Dsel* mRNA is most abundant in the head and is expressed at comparable levels in the dorsal and ventral trunk. **(B)** *Dse* expression. The bold lines indicate the level of sections in D and E. **(D-E’)** *Dse* expression is detected in the notochord, dermatome, sclerotome and hypochord (inset). **(C)** *Dsel* expression. The bold lines indicate the level of sections in F and G. **(F-G’)** *Dsel* expression appears in the ventral spinal cord, migrating trunk neural crest cells (arrowhead), myotome, sclerotome, hypochord (inset) and posterior dorsal hindgut endoderm. dm, dermatome; en, endoderm; hc, hypochord; my, myotome; no, notochord; sc, sclerotome; sp, spinal cord. Scale bars are 1 mm (B,C) and 200 μm (D-G’).

Using qPCR analysis, we quantified the *Dse* and *Dsel* mRNA levels in head, dorsal trunk and ventral trunk explants at stage 32 (Fig 10A). The *Dse* transcript levels were equal in these regions (Fig 10A top). The *Dsel* mRNA was 2-fold higher concentrated in the head than in the trunk, and comparable levels were found between the dorsal and ventral trunk region (Fig 10A bottom). Whole-mount *in situ* hybridization confirmed the relative distribution of both gene products in the head and dorsal trunk, but showed less intense staining in the ventral trunk (Fig 10B and 10C) likely as a consequence of quenching of *in situ* hybridization signals due to a high amount of yolk in the gut endoderm. A closer inspection of the trunk in transversally sectioned embryos (Fig 10D–10G’) revealed co-expression of *Dse* and *Dsel* in the hypochord and sclerotome cells that have migrated to the perineural and perinotochordal space [28]. Unique *Dse* expression was found in the notochord and dermatome (Fig 10D–10E’). *Dsel* expression alone appeared in the ventral hemisphere of the spinal cord, myotome and posteriorly in the dorsal hindgut endoderm (Fig 10F–10G’). Thus, *Dse* and *Dsel* share expression domains in the ventral brain, cranial neural crest, trigeminal (V) ganglia, sclerotome and hypochord.

## Discussion

The frog *Xenopus laevis* arose from hybridization of two diploid *Xenopus* species, leading to allotetraploidy with two related subgenomes [26]. The gene loci of the two dermatan sulfate epimerases are conserved between human [7] and *X*. *laevis* (this study), with the open reading frame of *Dse* spanning over five exons and that of *Dsel* confined to a single exon. DS-epi1 and DS-epi2 have a common epimerase domain and their enzymatic activities have been experimentally validated in human and *Xenopus* [7, 16, 27]. Three catalytic amino acids that were previously shown to be indispensable for the epimerase activity in human DS-epi1 are also present in human DS-epi2 and are conserved in the four *X*. *laevis* sequences, suggesting that each homeolog might contribute to the production of IdoA structures in CS/DS hybrid chains.

Our comparative analysis of the two DS biosynthetic enzymes reveals both common and differential expression of *Dse* and *Dsel* (Table 1). Previous RT-PCR results revealed maternal deposits of mainly *Dsel* mRNA in *Xenopus* blastula embryos [16]. Based on whole-mount *in situ* hybridization and qPCR analysis, we now show maternal expression of both genes with *Dsel* exhibiting higher mRNA levels than *Dse* at the 4-cell stage and at stage 9. We previously reported distinct expression domains of *Dse* in the epidermis, pre-migratory cranial neural crest and the notochord at stage 17 and of both *Dse* and *Dsel* in migratory cranial neural crest cells at stage 30 [16]. Here, we introduce a series of new expression domains. Partially overlapping expression of the two genes was observed in the adenohypophysis, eyes, brain, trigeminal (V) ganglia, sclerotome, and the dorsal endoderm (hypochord). Additional distinct *Dse* transcripts were found in the anterior surface ectoderm, spinal nerves, and dermatome, while *Dsel* was expressed in the spinal cord, epibranchial (VII, IX, X,) ganglia, prechordal mesendoderm and myotome. The relative contribution of the two DS epimerases to early IdoA production in CS/DS chains might differ in other vertebrates, since e.g. in zebrafish both *dse* and *dselb*, but not the duplicated *dsela* ortholog, are maternally expressed [18]. Similarly as in *Xenopus*, zebrafish *dse* is expressed in the eye and notochord, *dselb* in the midbrain/hindbrain boundary region, and both *dse* and *dselb* in the somites.

**Table 1:** Summary of gene expression for *Dse* and *Dsel* in *Xenopus laevis* embryo.

		cleavage	blastula	gastrula	neurula	tailbud	tadpole
		st. 1–6	st. 7–9	st. 10–13	st. 14–22	st. 23–40	st. 41-
**maternal**	***Dse***	**+**	**+**	** **	** **	** **	** **
	***Dsel***	**+++**	**+++**	** **	** **	** **	** **
**eye**	***Dse***	** **	** **	** **	** **	**+**	** **
	***Dsel***	** **	** **	** **	** **	**+**	** **
**forebrain**	***Dse***	** **	** **	** **	**+**	**+**	**-**
	***Dsel***	** **	** **	** **	**-**	**+**	**-**
**midbrain**	***Dse***	** **	** **	** **	**+**	**+**	**-**
	***Dsel***	** **	** **	** **	**-**	**+++**	**++**
**hindbrain**	***Dse***	** **	** **	** **	**+**	**+**	**-**
	***Dsel***	** **	** **	** **	**-**	**+++**	**++**
**spinal cord**	***Dse***	** **	** **	** **	**-**	**-**	**-**
	***Dsel***	** **	** **	** **	**-**	**++**	**-**
**cranial sensory ganglia**	***Dse***					**+**	
	***Dsel***	** **	** **	** **		**++**	
**spinal nerves**	***Dse***					**+**	
	***Dsel***					**-**	
**neural crest**	***Dse***	** **	** **	** **	**++**	**+++**	**++**
	***Dsel***	** **	** **	** **	**-**	**+++**	**-**
**anterior surface ectoderm**	***Dse***	** **	** **	** **	**++**	**++**	**-**
	***Dsel***	** **	** **	** **	**-**	**-**	**-**
**adenohypophysis**	***Dse***					**++**	
	***Dsel***					**+**	
**epidermis**	***Dse***	** **	** **	**+++**	**+++**	**++**	**-**
	***Dsel***	** **	** **	**-**	**-**	**-**	**-**
**prechordal mesendoderm**	***Dse***	** **	** **	** **	**+**	**-**	** **
	***Dsel***	** **	** **	** **	**-**	**++**	** **
**notochord**	***Dse***	** **	** **	**+**	**++**	**++**	**++**
	***Dsel***	** **	** **	**-**	**-**	**-**	**-**
**dermatome**	***Dse***	** **	** **	** **	** -**	**++**	** **
	***Dsel***	** **	** **	** **	** -**	**-**	** **
**myotome**	***Dse***	** **	** **	** **	** -**	**-**	** **
	***Dsel***	** **	** **	** **	** -**	**++**	** **
**sclerotome**	***Dse***	** **	** **	** **	**++**	**++**	** **
	***Dsel***	** **	** **	** **	**-**	**+**	** **
**dorsal endoderm**	***Dse***	** **	** **	** **	**++**	**++**	** **
	***Dsel***	** **	** **	** **	**-**	**++**	** **

Intensity levels based on whole-mount *in situ* hybridization are indicated as follows: -, no expression; +, weak; ++, intermediate; +++, strong. Note that not all stages were analyzed.

In *Xenopus*, *Dse* is dynamically expressed in distinct domains of the anterior surface ectoderm, including the anterior neural fold, sense plate and derived stomach anlage, adenohypophysis and hatching gland. Consistently, zebrafish *dsela* and *dselb* are expressed in mesoendodermal cells of the anterior polster [18]that gives rise to the hatching gland in teleosts. Hence the gene expression patterns of the DS-epimerases identifies the hatching gland as a possible site of CS/DS production and underscores its evolutionary origin from different germ layers in the frog and fish [33].

*Dse* shows abundant expression in the epidermis between stages 13 and 27 with low mRNA levels in the deep layer and higher levels in the superficial layer. The superficial epidermis is composed of polarized epithelial cells and abundantly expresses differentiation markers in contrast to the deep non-epithelial cells [34]. The bi-layered epidermis in *Xenopus* embryos is similar to the mammalian embryonic epidermis, which consists of an inner basal layer and a temporary outer periderm [35]. During mammalian skin development, basal stem cells divide and move to suprabasal locations, where they differentiate via a spinous cell intermediate to mature keratinocytes that eventually die and are shed from the skin surface [36]. Interestingly, newborn *Dse*-knockout mice have thickened basal and spinous layers of the epidermis with elevated levels of immature keratinocytes [13], supporting a positive role of DS-epi1 in epidermis differentiation.

*Dse* is dynamically expressed in the deep (sensorial) layer of the neural ectoderm. The site of expression changes from a lateral position in the open neural plate at stage 15 to a medial (then ventral) location in the invaginating neural groove at stage 18, where they stay until stage 32. Primary neurons develop in the sensorial layer and become post-mitotic in a wave, with Rohon-Béard sensory neurons differentiating first in lateral (then dorsal) positions followed by interneurons in intermediate and motoneurons in ventral positions of the closing neural tube [37, 38]. Hence, *Dse* expression appears to follow a spatio-temporal pattern in accordance with primary neuron differentiation. *Dsel* transcripts reach high levels between stages 27 and 43 in the inner mantle zone of the developing midbrain and hindbrain at sites where ventral interneurons differentiate. In tailbud embryos, *Dsel* and, to a lower extent, *Dse* are co-expressed in the trigeminal (V) ganglia. *Dsel* transcripts are also abundant in epibranchial ganglia, including the geniculate (VII), petrosol (IX) and nodose (X) ganglia, while *Dse* mRNA is transcribed in spinal nerves. Together, the gene expression of the two DS epimerases suggests that IdoA structures in CS/DS chains are produced at sites of neuronal cell differentiation in the developing nervous system.

Early in development, *Dse* is expressed in the epidermis and notochord from stage 13, in the neural plate, anterior neural ridge and prechordal mesendoderm from stage 15/16, and in the ventral brain primordia and somites from stage 18 onwards. These expression domains, together with *Dsel* gene activity in the ventral portion (alar plate) of the developing brain and spinal cord at tailbud stages are consistent with a possible contribution of CS/DS to neural tube closure. Knockout mice that lack either DS-epi1 alone (*Dse*-KO) or a combination of DS-epi1 and DS-epi2 (double-knockout, DKO) exhibit a low frequency (~5%) of exencephaly and spina bifida [13, 15]. Such neural tube abnormalities are common human birth defects and result from a failure to close the neural tube in the cranial region (exencephaly) or more caudally (spina bifida) due to misregulation of proliferation, differentiation and morphogenetic events in the neural plate as well as disturbed interaction with adjacent non-neural tissues [39].

In advanced tadpole embryos, *Dsel* expression was detected in ventral territories of the midbrain and anterior hindbrain, where dopaminergic and serotonergic neurons differentiate, respectively. Dopaminergic and serotonergic neurons are induced by morphogen signals that include FGF8 from the isthmic organizer and, in the case of serotonergic neurons, FGF4 from the notochord [40]. IdoA-containing CS/DS chains are known to bind and regulate FGF signals [41–43]. The expression of *Dse* and *Dsel* in the source (notochord) and/or receiving cells (mid- and hindbrain) of FGF signals might indicate a possible involvement of CS/DS in morphogen signaling during dopaminergic and serotonergic neuron differentiation. Our study supports previous studies in the postnatal mouse [44] and adult human [10] that *Dsel* is expressed at relatively high levels in the central nervous system. Two non-synonymous mutations in the coding region of *DSEL* (Y730C, I1113M) were reported in a heterozygous state in 3 individuals from a group of 113 bipolar disorder patients, but not in the control group [10]. Single nucleotide polymorphisms were also found upstream of *Dsel* in regions with possible regulatory function in early-onset major depressive disorder [11]. Since misregulation of dopamine and serotonine account for bipolar disorder and depression, a possible involvement of CS/DS in the differentiation or function of neuronal populations that express these monoamine neurotransmitters might help to explain the genetic link between human DSEL and these mood disorders.

While *Dse* is expressed in neural crest (NC) cells already at stage 15 and maintains expression therein at least until stage 40, *Dsel* is only transiently expressed in this migratory stem cell population between stages 27 and 34. Knockdown of *Dse* by injection of antisense oligonucleotides altered the expression of NC-specific genes and decreased the extent of NC cell migration, leading to reduction of craniofacial skeleton, lack of dorsal fin structures and reduced melanophores [16]. Indeed, transplantation experiments revealed a tissue-autonomous role of DS-epi1 in cranial NC cell migration and its indispensable role for cell adhesion, spreading and formation of polarized cell structures on fibronectin. These experiments suggested that the craniofacial abnormalities observed in MS-EDS patients might result from failure of cranial NC cell migration [17].

In the developing somites, *Dse* expression starts at stage 18 in the sclerotome and appears at stage 32 also in the dermatome, whereas *Dsel* expression at stage 32 is found in the sclerotome and myotome. The differential expression of DS epimerases in these paired blocks of paraxial mesoderm might correlate with previously reported phenotypic alterations in animal models and human conditions. First, the notochord induces the ventral portion of the somites to form the segmented sclerotome [45], which later gives rise to the axial skeleton (vertebrae, ribs). We suggest that reduced IdoA content in CS/DS chains in the notochord and sclerotome not only relates to the kinked tail phenotype in *Dse*-morphant *Xenopus* tadpoles [16] as well as *Dse*-KO and DKO mouse embryos and pups [12–15], but also explains the spinal and chest wall deformities in MS-EDS patients [46]. Second, decreased levels of DS-epi1 in the dermatome might contribute to lowered tensile strength of the skin and the abdominal wall closure defect in *Dse*-KO mice [12, 13] and tissue fragility involving the skin, joints and multiple organs in MS-EDS [46].

Thus, by mapping the spatio-temporal gene expression of two genes that are essential for IdoA biosynthesis on CS/DS proteoglycans, we show that clinical manifestations of MS-ESD and psychiatric disorders are correlated with sites where *Dse* and *Dsel* are expressed.

## Conclusions

The expression of *Dse* and *Dsel* in the early *Xenopus* embryo coincides with sites of cell differentiation in the epidermis and neural tissue. Their expression in the ectoderm and underlying notochord and somites might account for the failed neural tube closure (exencephaly and spina bifida), which was reported in knockout mice. Robust expression of *Dsel* in the developing midbrain and anterior hindbrain, where dopaminergic and serotonergic neurons differentiate, might help to explain the genetic link of *DSEL* to bipolar disorder and depression. Several expression domains of *Dse* can be associated with congenital defects in the musculocontractural Ehlers-Danlos syndrome, such as the sclerotome to spinal/chest wall deformities and the dermatome to skin fragility.

## Supporting information

S1 Fig Epimerase domains of DS-epi1 and DS-epi2. The alignment was performed using ClustalW (EMBL-EBI) and BoxShade (ExPASy). The catalytic residues His205, Tyr261 and His450 in DS-epi1 are indicated with stars and are also conserved in DS-epi2. The total amino acid number and the percentage of amino acid identity to DS-epi1.S are indicated at the end of each sequence. Accession numbers of the *Xenopus laevis* protein sequences are: DS-epi1.S, KU877109; DS-epi1.L, XM_018263281; DS-epi2.S, XM_018223616; DS-epi2.L, KU877110.
(TIF)
