# Supplementary figures and images for: Gene expression of the two developmentally regulated dermatan sulfate epimerases in the Xenopus embryo
Source: PLoS One. 2018 Jan 25;13(1):e0191751. doi: 10.1371/journal.pone.0191751 (PMC5784981; doi:10.1371/journal.pone.0191751)

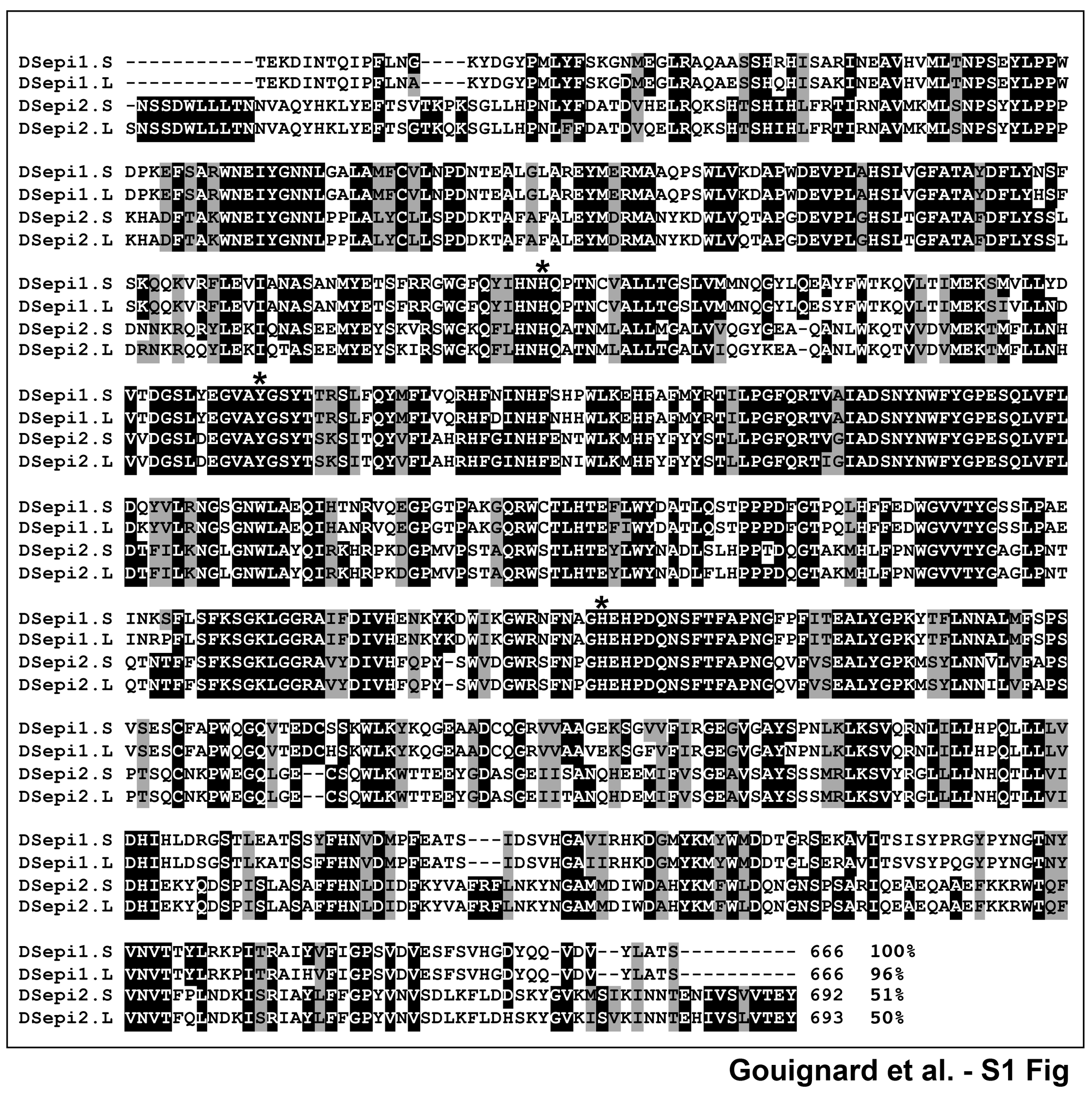

Supplement: S1 Fig — The alignment was performed using ClustalW (EMBL-EBI) and BoxShade (ExPASy). The catalytic residues His205, Tyr261 and His450 in DS-epi1 are indicated with stars and are also conserved in DS-epi2. The total amino acid number and the percentage of amino acid identity to DS-epi1.S are indicated at the end of each sequence. Accession numbers of the Xenopus laevis protein sequences are: DS-epi1.S, KU877109; DS-epi1.L, XM_018263281; DS-epi2.S, XM_018223616; DS-epi2.L, KU877110. (TIF) [file pone.0191751.s001.tif]
